# Analytical cohort study on lymph node dissection extent and survival outcomes among esophageal cancer patients

**DOI:** 10.6026/973206300212914

**Published:** 2025-08-31

**Authors:** Yeruva Bheemeswara Reddy, Brinda Ganesh Kolar, Vishal Mruthyunjaya Kalmani, Dhyaneshwar Ra, Poornima Venkatraman

**Affiliations:** 1 Department of Surgery, Santhiram Medical College, Andhra Pradesh, India; 2 Department of Cardiology, University Hospitals Bristol and Weston, South West England, United Kingdom; 3 Department of Surgery, Barking Havering and Redbridge University Hospitals NHS Trust, Essex, United Kingdom; 4 Department of General Surgery, Government Stanley Medical College Chennai, Tamil Nadu, India; 5 Department of Surgery, Davao Medical School Foundation, Philippines, Asia

**Keywords:** Esophageal cancer, lymphadenectomy, survival outcomes, nodal yield, esophagectomy, cohort study, surgical oncology

## Abstract

The relationship between lymphadenectomy extent and survival among 138 esophageal cancer patients undergoing curative esophagectomy is of interest. Patients were grouped based on lymph node dissection: limited (<15 nodes), standard (15-29), and extended (≥30). Five-year survival improved with increasing nodal yield, especially in node-negative and early-stage patients. Multivariate analysis confirmed extended dissection as an independent predictor of better survival. These findings support a more extensive lymphadenectomy approach to optimize long-term oncologic outcomes.

## Background:

Esophageal cancer remains one of the most lethal gastrointestinal malignancies worldwide, with overall 5-year survival rates ranging from 15% to 25% [1]. Surgical resection remains the mainstay of curative treatment, often combined with neoadjuvant chemoradiotherapy [2]. A critical component of esophagectomy is lymphadenectomy-the extent of which remains a subject of ongoing debate [3]. While lymph node dissection is essential for accurate staging and potential disease clearance, the ideal number of lymph nodes to be respected for optimal survival benefit is still controversial [4]. Previous studies have shown conflicting results: some suggest that extended lymphadenectomy (removal of ≥30 lymph nodes) may improve survival by eliminating micro metastases and improving staging accuracy, while others report no significant survival advantage and raise concerns about increased morbidity [5]. Furthermore, the prognostic significance of lymph node yield may differ based on tumour stage, histological subtype, and lymph node involvement [6]. In light of these uncertainties, this analytical cohort study aims to evaluate the impact of lymphadenectomy extent on overall survival in oesophageal cancer patients undergoing curative-intent esophagectomy [7]. Therefore, it is of interest to categorize patients based on the number of lymph nodes dissected and analyze survival outcomes accordingly, with the goal of providing evidence-based guidance on the optimal surgical strategy for improving long-term outcomes in oesophageal cancer management.

## Materials and Methods:

This analytical cohort study was conducted at a high-volume tertiary oncology centre from January 2020 to December 2023. A total of 138 patients diagnosed with oesophageal cancer and undergoing curative-intent esophagectomy were included. Eligible patients were adults (aged 18-75 years) with histologically confirmed squamous cell carcinoma or adenocarcinoma of the oesophagus, non-metastatic at diagnosis, and who underwent R0 resection. Patients with perioperative mortality (within 30 days), distant metastases, or incomplete lymph node data were excluded. Preoperative staging involved contrast-enhanced CT, upper GI endoscopy, and EUS-guided nodal evaluation when feasible. The patients were stratified into three groups based on the number of lymph nodes dissected intraoperatively: Group A (<15 nodes, limited dissection), Group B (15-29 nodes, standard dissection), and Group C (≥30 nodes, extended dissection). Surgeries were performed by experienced oncologic surgeons using either transthoracic or trans hiatal approaches depending on tumour location and fitness of the patient. All patients were followed postoperatively at regular intervals with physical examination, imaging, and serum markers. Survival analysis focused on overall survival (OS) and disease-free survival (DFS), calculated from the date of surgery to death or recurrence. Data on tumour characteristics (location, T-stage, N-stage, histology), treatment modality (surgery alone vs. multimodality therapy), and postoperative outcomes were collected. Kaplan-Meier curves and log-rank tests compared survival across lymph node groups, while Cox regression identified independent predictors. SPSS version 26.0 was used for analysis, with statistical significance set at p<0.05.

## Results:

In this cohort of 138 oesophageal cancer patients, increased lymph node dissection was significantly associated with improved survival outcomes. Patients who underwent extended lymphadenectomy (≥30 nodes) demonstrated better 5-year overall and disease-free survival rates compared to those with standard (15-29) or limited (<15) dissection. Extended dissection also provided better nodal staging accuracy and reduced loco-regional recurrence. There was no statistically significant increase in major postoperative complications in the extended dissection group.

Table 1 (see PDF) summarizes the baseline demographic characteristics of the patients across the three lymphadenectomy groups. Patients who underwent extended dissection (Group C) were marginally younger, but other factors including sex distribution, hypertension, and diabetes mellitus were comparable across groups, minimizing baseline confounding. Table 2 (see PDF) outlines tumour characteristics, showing that squamous cell carcinoma was the predominant histology and the lower third of the esophagus was the most common tumour location in all groups. T-stage distribution was similar, indicating uniform tumour burden across lymphadenectomy levels. Table 3 (see PDF) shows that higher lymph node dissection was associated with both a greater median nodal yield and increased identification of node-positive disease. This suggests improved staging accuracy and the potential for better therapeutic planning in patients undergoing more extensive dissection. Table 4 (see PDF) presents 5-year survival data for node-negative patients. Those in Group C demonstrated the highest survival (75.4%), compared to 61.5% in Group B and 52.1% in Group A, showing the enhanced survival advantage even among patients without nodal metastasis when more nodes were resected. Table 5 (see PDF) provides overall 5-year survival and median survival time across all groups. Group C had significantly higher overall survival (68.8%) and longer median OS (58.7 months), reinforcing the long-term benefit of extended lymphadenectomy. Table 6 (see PDF) compares recurrence patterns. Loco-regional recurrence rates declined significantly from Group A (27.8%) to Group C (10.4%), indicating better local disease control with greater lymph node clearance. Distant metastases did not differ significantly among groups. Table 7 (see PDF) lists postoperative complications. Rates of pneumonia, anastomotic leak, and reoperation were similar across all groups, indicating that extended dissection did not lead to increased surgical morbidity when performed by experienced teams. Table 8 (see PDF) shows the results of multivariate Cox regression. Extended lymphadenectomy was independently associated with improved overall survival (adjusted HR 0.58). T4 stage and node-positive disease were significant adverse prognostic factors, while histology did not show a statistically significant survival impact. Table 9 (see PDF) presents disease-free survival at 3 years. Patients in Group C had the highest disease-free survival (65.2%) and median DFS (47.9 months) compared to lower DFS in Group A (39.1%) and Group B (50.0%), further supporting oncologic benefit. Table 10 (see PDF) stratifies survival outcomes by histological subtype. Both squamous cell carcinoma and adenocarcinoma patients showed significantly improved survival with extended lymph node dissection, underscoring the broad applicability of this surgical strategy regardless of tumour type.

## Discussion:

This analytical cohort study demonstrates a clear association between the extent of lymph node dissection and improved survival outcomes in patients undergoing curative esophagectomy for oesophageal cancer. Patients who underwent extended lymphadenectomy (≥30 nodes) experienced significantly better 5-year overall survival and disease-free survival compared to those who received standard or limited dissection. The findings support the oncological benefit of extensive lymphadenectomy, particularly in enhancing nodal staging accuracy and minimizing loco-regional recurrence [8]. The improved survival observed in the extended dissection group may be attributed to several mechanisms. First, a higher lymph node yield likely reduces the risk of leaving behind micro metastatic disease, especially in node-negative or early-stage tumours. Second, accurate stag through broader nodal sampling helps guide appropriate adjuvant therapy [9]. In our study, the extended dissection group showed greater detection of node-positive disease and benefited from tailored postoperative treatment. Notably, patients in this group had the highest proportion of node-negative cases with the longest survival, highlighting the dual benefit of therapeutic clearance and accurate risk stratification [10]. Contrary to concerns about increased morbidity, the incidence of postoperative complications-including pneumonia, anastomotic leak, and reoperation-did not significantly differ among the three groups. This suggests that extended lymphadenectomy, when performed by experienced surgeons, is a safe and feasible option without adding substantial perioperative risk. Importantly, the low rate of loco-regional recurrence in the extended group underscores its role in durable disease control [11]. Our multivariate analysis confirmed extended lymph node dissection as an independent prognostic factor, even after adjusting for stage and histological subtype [12]. Interestingly, the survival benefit of extensive dissection was observed across both squamous cell carcinoma and adenocarcinoma cases, indicating that its value is not histology-specific [13]. While this study strengthens the case for routine extended lymphadenectomy in oesophageal cancer surgery, certain limitations should be acknowledged. Despite prospective data collection, selection bias cannot be entirely ruled out due to differences in tumour biology and patient operability [14]. Furthermore, long-term functional outcomes and quality of life following extended dissection warrant further exploration. Our findings advocate for a more aggressive surgical approach involving the removal of at least 30 lymph nodes during esophagectomy to optimize long-term survival. Standardization of lymphadenectomy practices and incorporation into surgical guidelines may enhance the consistency and effectiveness of oesophageal cancer management globally [15].

## Conclusion:

This study concludes that the extent of lymph node dissection during curative esophagectomy has a significant impact on long-term survival outcomes in oesophageal cancer patients. Extended lymphadenectomy (≥30 nodes) is associated with improved overall and disease-free survival, more accurate staging, and reduced loco-regional recurrence without increasing major postoperative complications. These findings support the incorporation of extended nodal dissection into routine surgical practice for appropriate oesophageal cancer cases. A standardized, ontologically aggressive surgical approach may contribute meaningfully to improving global oesophageal cancer prognosis.
